# An engineered and scalable human cell-derived extracellular matrix demonstrates advantages over recombinant collagen in biomimicry and wound healing

**DOI:** 10.3389/fbioe.2026.1892416

**Published:** 2026-07-29

**Authors:** Xiaolei Guo, Xiaoye Ran, Yi Zhang, Boxun Liu, Zhengbo Wen, Yuanbo Liu, Mengqi Zhou, Mengqing Zang, Jiadao Wang, Tao Xu

**Affiliations:** 1 State Key Laboratory of Tribology, Tsinghua University, Beijing, China; 2 Center for Medical Device Evaluation, National Medical Products Administration, Beijing, China; 3 Department of Plastic and Reconstructive Surgery, Plastic Surgery Hospital, Chinese Academy of Medical Sciences and Peking Union Medical College, Beijing, China; 4 Department of Research and Development, Huaqing Zhimei (Shenzhen) Biotechnology Co., Ltd., Shenzhen, China; 5 Bio-Intelligent Manufacturing and Living Matter Bioprinting Center, Research Institute of Tsinghua University in Shenzhen, Tsinghua University, Shenzhen, China

**Keywords:** biomimetic biomaterials, human cell-derived engineered extracellular matrix, recombinant collagen, skin regeneration, wound healing

## Abstract

Skin wound healing remains a major clinical challenge due to impaired regeneration and incomplete restoration of dermal architecture, creating the need for biomaterials that reconstruct the human extracellular matrix (ECM) microenvironment, especially the collagen-rich structural network. Current strategies to obtain human-relevant collagen primarily include genetically engineered recombinant collagen and ECM directly produced by human cells. Although recombinant collagen has been extensively developed, it represents a simplified surrogate of native human collagen, and the biosynthesis of fully mature human collagen remains technically challenging. Therefore, we developed an engineered and scalable strategy to fabricate human cell-derived engineered ECM (HE-ECM) from human fibroblasts and systematically compared it with commercial recombinant collagen. Physicochemical analyses demonstrated that HE-ECM preserved native-like porous matrix architecture, collagen triple-helical integrity, enhanced thermal stability, and a more complex protein composition. HE-ECM significantly enhanced fibroblast viability, proliferation, and migration *in vitro* and accelerated wound closure with improved tissue organization in a rat full-thickness skin defect model. Overall, this controllable and donor-independent fabrication strategy enables scalable production of human collagen-rich HE-ECM that more faithfully recapitulates the native skin microenvironment than recombinant collagen, thereby suggesting its potential as a biomimetic platform for tissue regeneration.

## 1 Introduction

Skin wounds, such as diabetic ulcers, burns, and traumatic defects, remain a major clinical challenge due to prolonged inflammation, impaired angiogenesis, and excessive scar formation (Sen et al., 2009; Rowan et al., 2015). Although conventional treatments, including debridement, negative pressure therapy, skin grafts, and growth factor application, can accelerate healing to some extent, they often fail to achieve complete regeneration of skin appendages and natural dermal architecture, resulting in variability of healing effects (Eriksson et al., 2022). Therefore, the development of biomaterials that can actively regulate the wound microenvironment and promote efficient healing has become a key goal in regenerative medicine.

Extracellular matrix (ECM) plays a central role in maintaining tissue architecture and regulating cell-matrix interactions, with collagen serving as its principal structural component (Avila Rodríguez et al., 2018; Naomi et al., 2021). Owing to the excellent biocompatibility and ability to support cell adhesion and matrix remodeling, collagen has long been regarded as a cornerstone of biomaterials in tissue engineering and regenerative medicine, where it is widely applied in wound healing and skin regeneration (Sionkowska et al., 2020; Davison-Kotler et al., 2019). Traditional collagen is primarily derived from animal sources such as bovine, porcine, and marine tissues. However, these sources raise concerns regarding immunogenicity and potential xenogeneic immune responses due to residual non-human proteins, as well as pathogen transmission, batch-to-batch variability, and the difficulty of completely removing protein impurities (Guo et al., 2024). To obtain collagen materials that are safer and closer to human physiology, recombinant collagen has been developed through genetic engineering approaches in microbial, mammalian, or plant-based expression systems. This strategy aims to produce human collagen sequences with improved purity, safety, and batch consistency compared with animal-derived collagen (Guo et al., 2024; Cheng et al., 2024). However, despite these advantages, recombinant collagen remains a simplified approximation of native human collagen. It still lacks complete post-translational modifications, exhibits lower molecular weight and weaker fibril assembly, undergoes rapid degradation, and fails to fully reproduce the complex biochemical cues of the native ECM (Cao et al., 2024; He et al., 2025; O'Donnell et al., 2019). As a result, its ability to faithfully reconstruct the human wound microenvironment and support long-term tissue regeneration remains limited.

To more accurately reproduce the composition and architecture of human ECM, an alternative strategy has emerged based on ECM directly produced by human cells *in vitro* (Assunção et al., 2020). Human cell-derived engineered ECM (HE-ECM) is obtained by culturing human cells to deposit ECM proteins, most notably human collagen, followed by decellularization to remove cellular components while preserving structural proteins, glycosaminoglycans, growth factors, and native-like fibrillar networks (Xu et al., 2024). Compared with recombinant collagen, HE-ECM better recapitulates the complexity of the natural microenvironment, particularly the native structural organization and biochemical context of human collagen, thereby enhancing cell adhesion, proliferation, migration, and lineage-specific differentiation (Ahlfors and Billiar, 2007). Recent studies have demonstrated its potential in wound repair, stem cell expansion, and tissue or organ reconstruction, suggesting that HE-ECM may serve as a promising biomimetic scaffold for regenerative applications (Suhaeri et al., 2018; Lin et al., 2012; Bourget et al., 2012). Despite these advances, the production of HE-ECM from small-scale cell cultures still faces critical challenges, including ethical constraints, donor-to-donor variability, limited availability, and the lack of standardized manufacturing protocols (Xu et al., 2024).

**FIGURE 1:**
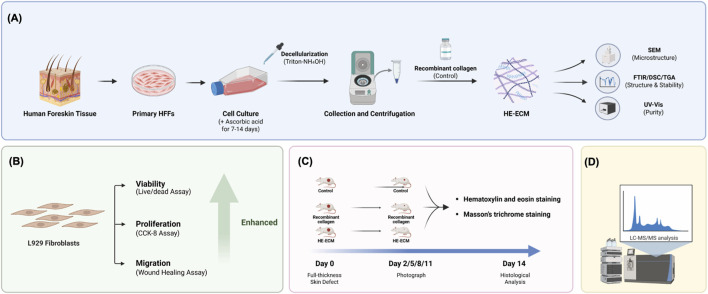
Schematic overview of the study design and experimental workflow. The overall experimental strategy is illustrated, including: **(A)** HE-ECM fabrication and characterization, **(B)** *In vitro* evaluation, **(C)** *In vivo* wound healing assessment, and **(D)** proteomic profiling. Created with BioRender.com.

To overcome these limitations, we established a scalable and engineered fabrication strategy for HE-ECM using human fibroblasts cultured *in vitro*. This fabrication method enables controllable, reproducible, and cell-line-based production of ECM with preserved ultrastructure and biochemical composition, providing a feasible solution for large-scale clinical application. Moreover, despite increasing interest in both recombinant collagen and HE-ECM, direct and systematic comparisons between these two strategies remain scarce. As both approaches aim to obtain collagen materials suitable for human tissue repair, clarifying their relative physicochemical and biological performance is essential for rational biomaterial selection. In this study, we systematically compared HE-ECM produced using our method with commercial recombinant collagen from the perspective of wound repair through both *in vitro* and *in vivo* experiments (Figure 1). Our findings indicate that HE-ECM more closely mimics the native skin microenvironment, enhances fibroblast activity, and significantly accelerates wound closure and collagen remodeling *in vivo* compared with recombinant collagen. Our study not only established a reproducible and engineered strategy for the large-scale production of HE-ECM but also demonstrated its notable regenerative potential, providing an evidence-based framework for optimizing biomaterial selection in tissue engineering and clinical wound management.

## 2 Materials and methods

### 2.1 Cell culture

Human foreskin fibroblasts (HFFs) were purchased from the Cell Bank of the Chinese Academy of Sciences (Shanghai, China). Cells were cultured in Dulbecco’s modified Eagle medium (DMEM) (Gibco, 11995-065) supplemented with 10% fetal bovine serum (FBS) (Gibco, 10099-141C) and 1% penicillin-streptomycin (Gibco, 15140-122) at 37 °C with 5% CO_2_. When cells reached 70%–80% confluence, they were trypsinized and subcultured to culture flasks. Fibroblasts at passage three to five (P3-5) were used for subsequent experiments.

### 2.2 Fabrication of HE-ECM

For HE-ECM fabrication, HFFs were cultured in 15-cm culture dishes. When the HFFs reached 80%–90% confluence, the medium was replaced with fresh culture medium supplemented with ascorbic acid (25 μg/mL) (Macklin, A800296) to promote ECM synthesis. The medium was refreshed every 2 days to maintain nutrient availability. After induction for 10 days, the medium was discarded, and the cultures were washed three times with PBS. For decellularization, 3 mL of 0.5% Triton-NH_4_OH solution (Leagene, PS0005) was added to each 15-cm dish, followed by incubation in a CO_2_ incubator for 3–5 min until complete cell detachment was achieved. The solution was subsequently removed, and the dishes were washed three times with PBS. The remaining material on the dish surface was gently scraped using a cell scraper and centrifuged at 1,000 rpm for 10 min at 4 °C. The resulting pellet was collected as the HE-ECM. The ECM samples were lyophilized prior to subsequent experiments.

### 2.3 Assessment of decellularization efficiency

HFF-derived ECM samples before and after decellularization were observed under a light microscope to assess cell removal. For nuclear staining, samples were fixed with 4% paraformaldehyde, washed with PBS, and stained with DAPI for 5 min at room temperature. After additional PBS washes, the samples were imaged using a fluorescence microscope to detect residual nuclei.

Residual DNA content in HE-ECM was quantified using a magnetic bead-based DNA purification method followed by PicoGreen dsDNA fluorescence detection. Lyophilized HE-ECM samples were weighed, and residual DNA was extracted using a magnetic bead-based DNA sample preparation kit (BeYoMag™ Magnetic Bead-Based DNA Sample Preparation Kit; Beyotime Biotechnology, China). The extracted DNA was quantified using a PicoGreen dsDNA assay kit (Quant-iT™ PicoGreen® dsDNA Assay Kit; Thermo Fisher Scientific, United States) with a microplate reader. Residual DNA content was normalized to the dry weight of HE-ECM and expressed as ng/mg dry ECM.

### 2.4 Quantification of collagen and total carbohydrate content

Protein profiles of HE-ECM were analyzed by SDS-PAGE followed by Coomassie brilliant blue staining. Lyophilized HE-ECM was dissolved in sample buffer, denatured, and loaded onto polyacrylamide gels together with a protein molecular weight marker. After electrophoresis, gels were stained with Coomassie brilliant blue and destained until clear protein bands were visible. Bovine type I collagen was used as a collagen reference control.

Collagen content in HE-ECM was determined by hydroxyproline quantification. Briefly, lyophilized HE-ECM samples were hydrolyzed in 6 M HCl at 105 °C for 24 h under nitrogen. After cooling, the hydrolysates were neutralized with NaOH, diluted to a defined volume, and subjected to colorimetric detection using an oxidizing reagent and chromogenic reagent. Absorbance was measured at 560 nm, and hydroxyproline concentration was calculated based on an L-hydroxyproline standard curve. Collagen content was then calculated using a hydroxyproline-to-collagen conversion factor of 7.46.

Total carbohydrate content in HE-ECM was determined using the phenol-sulfuric acid colorimetric method. Briefly, HE-ECM samples were dissolved in 0.5 M acetic acid to prepare sample solutions. D-glucose standard solutions were used to generate a standard curve. Blank, standard, and sample solutions were mixed with phenol solution and concentrated sulfuric acid, followed by incubation at 40 °C. After cooling, absorbance was measured at 490 nm. Total carbohydrate content was calculated from the D-glucose standard curve and expressed as D-glucose equivalents.

### 2.5 Scanning electron microscopy (SEM) observation

The microstructure of HE-ECM and recombinant collagen (type I collagen, Juyuan Biotech Co., Ltd.) was characterized using SEM (FEI Quanta 450, SU3500). The lyophilized samples were mounted on specimen stubs and sputter-coated with gold (sputtering current: 20 mA; duration: 60 s; coating thickness: 5 nm) to enhance surface conductivity. The surface morphology was examined using SEM operated at an accelerating voltage of 3–15 kV and a working distance of 7–15 mm. Representative images were acquired from at least three randomly selected regions.

### 2.6 Fourier transform infrared (FTIR) spectroscopy

The chemical structure of the samples was analyzed using FTIR spectroscopy (Thermo Fisher Scientific Nicolet iN10). The lyophilized samples were finely ground with potassium bromide at a mass ratio of 1:100 and pressed into transparent pellets. Spectra were recorded using an infrared spectrophotometer over the range of 4,000–400 cm^−1^ with a resolution of 4 cm^−1^ and 32 scans. Baseline correction and spectral smoothing were performed using Origin software, and characteristic absorption peaks were subsequently identified.

### 2.7 Differential scanning calorimetry (DSC)

To evaluate the thermal stability of collagen, DSC (Netzsch, Germany) was employed to determine the denaturation temperature (Tm) and enthalpy change (ΔH) of recombinant collagen and HE-ECM. Accurately weighed samples (5 mg) were sealed in aluminum pans, with an empty pan used as the reference. The samples were heated from 25 °C to 250 °C at a rate of 10 °C/min under a nitrogen atmosphere, and the heat flow was continuously recorded. The characteristic endothermic peaks corresponding to denaturation temperature (Tm, 40 °C–70 °C) and decomposition (Tm, >200 °C) were analyzed to compare the thermal stability of recombinant collagen and HE-ECM.

### 2.8 Ultraviolet–visible (UV–Vis) absorption spectroscopy

The characteristic protein absorption profiles were assessed by ultraviolet–visible (UV–Vis) spectroscopy (PE lambda 750). Lyophilized samples were dissolved in 0.1 M acetic acid, centrifuged to remove insoluble residues, and appropriately diluted to obtain absorbance values within the linear detection range. UV–Vis spectra were recorded over the wavelength range of 200–400 nm. Characteristic absorption peaks corresponding to peptide bonds and aromatic amino acid residues were analyzed to compare the protein composition and structural characteristics of recombinant collagen and HE-ECM.

### 2.9 Thermogravimetric analysis (TGA)

TGA was performed to evaluate the thermal degradation behavior (NETZSCH TG 209 F1 Libra, Germany). Samples were vacuum-dried to constant weight to eliminate residual moisture. Approximately 5 mg of each sample was placed in an alumina crucible and heated from room temperature to 800 °C at a heating rate of 5 °C/min under an air atmosphere. The purge and protective gas flow rates were set at 20 mL/min according to the instrument program. Changes in sample mass were continuously recorded as a function of temperature, and thermogravimetric curves were obtained after normalization to the initial sample mass. Derivative thermogravimetry (DTG) curves were calculated from the first derivative of the TGA curves to identify the major stages of thermal degradation. Residual mass and characteristic degradation behavior were analyzed to compare the thermal stability of recombinant collagen and HE-ECM.

### 2.10 Circular dichroism (CD) spectroscopy

CD spectroscopy was performed to evaluate the secondary structural features of recombinant collagen and HE-ECM. Lyophilized recombinant collagen and HE-ECM samples were dissolved in PBS at the same concentration and transferred to a quartz cuvette. Far-UV CD spectra were recorded over a wavelength range of 190–260 nm at room temperature. The CD signal was collected as a function of wavelength, and the spectra were used to compare the collagen-related secondary structural features of the two samples.

### 2.11 Cell viability assessment

The effects of recombinant collagen and HE-ECM on cell viability were evaluated using a fluorescent Live/Dead assay and the Cell Counting Kit-8 (CCK-8) assay (n = 3 per group). For the Live/Dead assay, passage-3 L929 fibroblasts (purchased from Shenzhen TuoTaiNuo Biotechnology Co., Ltd., China) were seeded into 48-well plates at 1 × 10^5^ cells/mL and cultured until 80%–90% confluence. L929 fibroblasts were selected for *in vitro* evaluation as a standardized cell model widely recommended for cytotoxicity and biomaterial screening (ISO 10993 guidelines). These assays were intended as preliminary and reproducible cytocompatibility and migration screening rather than human-specific functional validation. The samples were then added for co-culture, with wells without samples serving as the blank control. After 24 h, the medium was removed, and the cells were washed three times with PBS. Subsequently, 200 μL of Calcein-AM/PI solution (Beyotime, C1371) was added and incubated at 37 °C in the dark for 15 min. Fluorescence images of live (green) and dead (red) cells were obtained using a fluorescence microscope (Olympus BX53, Japan), and five random fields per well were imaged. The percentage of live cells was quantified using ImageJ software.

For the CCK-8 assay, L929 fibroblasts at passage 3 were seeded into 96-well plates at 2 × 10^3^ cells per well and incubated for 24 h. The samples were then added for co-culture. At 24 h and 48 h, the cells were washed three times with PBS, followed by the addition of 10 μL of CCK-8 working solution (Beyotime, C0038) (CCK-8: DMEM = 1:9, v/v). After incubation for 2 h at 37 °C in the dark, 100 μL of supernatant from each well was transferred to a new 96-well plate, and absorbance at 450 nm was measured with a microplate reader. Cells cultured in DMEM containing 10% FBS served as the positive control (100% viability). Cell viability was calculated using the following equation:

$$\text{Cell viability }\left(\%\right)=\left(A_{e}-A_{n}\right)/\left(A_{p}-A_{n}\right)\times 100$$

where A_p_ is the absorbance of the positive control group, A_n_ is the absorbance of the negative control group, and A_e_ is the absorbance of the experimental group.

### 2.12 Migration assay

The effects of recombinant collagen and HE-ECM on cellular motility were evaluated using a scratch assay (n = 3 per group). L929 fibroblasts in the logarithmic growth phase were seeded into 6-well plates at a density of 5 × 10^5^ cells/mL. When the cells reached 80%–90% confluence, the medium was removed and washed three times with PBS and starved with FBS-free medium for 18 h. Subsequently, a straight scratch was created across the center of each well using a sterile 200 μL pipette tip. The culture medium was discarded, and the wells were washed three times with PBS to remove detached cells. Serum-free DMEM supplemented with either recombinant collagen or HE-ECM samples was then added, and the plates were incubated under standard conditions. Images of the scratch areas were captured at 0 h and 12 h and analyzed using ImageJ software. Cell migration was quantified by calculating the relative cell migration rate using the following equation:

$$\text{Migration rate }\left(\%\right)=\left(N_{0}-N_{12}\right)/N_{0}\times 100\%$$

where N_0_ is the initial wound area and N_12_ is the remaining area at 12 h.

### 2.13 *In vivo* wound healing

All animal experiments were carried out under the guidance of the Medical Ethics Committee of Plastic Surgery Hospital, Chinese Academy of Medical Sciences and Peking Union Medical College (Approval no. EAEC 2024-026). Eight-week-old male Sprague-Dawley (SD) rats (purchased from Zhuhai BesTest Bio-Tech Co., Ltd.) were randomly divided into three groups (n = 6 per group, based on previous wound-healing studies and experimental feasibility): blank control (treated with normal saline), recombinant collagen (10 mg/mL), and HE-ECM (10 mg/mL). Recombinant collagen and HE-ECM working suspensions were prepared at 10 mg/mL by dissolving 10 mg of lyophilized material in 1 mL of PBS. Following anesthesia with isoflurane, the dorsal hair of each rat was shaved, and the skin was disinfected with povidone-iodine. A full-thickness excisional wound with a diameter of 1 cm was created on the dorsal skin using a sterile biopsy punch. Subsequently, 0.5 mL of the corresponding treatment was topically applied dropwise to the wound site. The wounds were then covered with 3M Tegaderm and gauze. Each rat was housed separately to prevent interference with wound dressings. The dressings were replaced every 2 days, and the wound appearance was photographed on day 0, 2, 5, 8, 11, and 14. The wound area was quantified using ImageJ software and the wound healing rate was calculated using the following equation:

$$\text{Remaining wound area }\left(\%\right)=\text{residual size}/\text{original size}\times 100\%$$

On day 14, the animals were euthanized by CO_2_ inhalation, and wound tissues were harvested for histological analysis.

### 2.14 Histological analysis

Hematoxylin and eosin (H&E) staining and Masson’s trichrome staining were used to evaluate tissue formation and assess collagen deposition, respectively. Wound tissues were fixed in 4% paraformaldehyde, dehydrated through a graded ethanol series, and embedded in paraffin. Sections were cut perpendicular to the wound surface and subjected to routine H&E staining and Masson’s trichrome staining. Images were captured using a light microscope and analyzed using ImageJ software.

### 2.15 Proteomic analysis

Proteomic analysis was performed on three biological replicates of HE-ECM samples by Beijing Biotech Pack Scientific Co., Ltd. (Beijing, China). Briefly, the samples was analyzed by nanoLC–MS/MS using an Easy-nLC 1,200 system coupled to an Orbitrap Exploris 480 mass spectrometer (Thermo Fisher Scientific, United States). Proteins were separated by SDS–PAGE, digested with trypsin, and the resulting peptides were analyzed on a NanoViper C18 column (75 μm × 25 cm). MS data were acquired in data-dependent mode with full scans at a resolution of 120,000 (m/z 300–1,800), followed by MS/MS of the top 20 most intense precursor ions. Raw data were processed using MaxQuant (version 1.6.2.10) and searched against a species-specific protein database. Carbamidomethylation of cysteine was set as a fixed modification, oxidation of methionine and N-terminal acetylation as variable modifications, with trypsin specified as the digestion enzyme and up to two missed cleavages allowed. Both precursor and fragment mass tolerances were set to 20 ppm, and the significance threshold was set at 0.01. To characterize the ECM composition of HE-ECM, the identified proteins were further annotated using Matrisome AnalyzeR based on the MatrisomeDB database (Petrov et al., 2023). Proteins were classified into core matrisome categories, including collagens, ECM glycoproteins, and proteoglycans, as well as matrisome-associated categories, including ECM regulators, ECM-affiliated proteins, and secreted factors. Representative ECM-related proteins were selected based on their ECM relevance and signal intensity, and were grouped into collagen/structural ECM proteins, cell-matrix interaction-related proteins, and collagen-processing or ECM-remodeling-associated proteins. To further interpret the biological relevance of the identified proteins, functional enrichment analysis was performed using the Metascape platform. Kyoto Encyclopedia of Genes and Genomes (KEGG) pathway enrichment analysis was conducted as exploratory pathway annotation of the HE-ECM proteome.

### 2.16 Statistical analysis

Data are expressed as means ± standard deviation (SD) (n ≥ 3). Statistical analysis was performed using GraphPad Prism version 10.0 software. Normality was assessed before parametric testing. For normally distributed data, comparisons between two groups were performed using an independent-samples *t*-test, while multiple-group comparisons were analyzed by one-way analysis of variance (ANOVA) followed by *post hoc* multiple-comparison tests. For data that did not meet normality assumptions, non-parametric tests were applied. A *P* value <0.05 was considered statistically significant.

## 3 Results

### 3.1 Decellularization validation and biochemical composition of HE-ECM

For batch preparation, up to 500 15-cm culture dishes were processed in parallel under the same induction and decellularization conditions. This workflow yielded approximately 125 g of wet HE-ECM per batch before lyophilization, with an average yield of approximately 0.25 g per dish. The decellularization efficiency of HE-ECM was evaluated by bright-field imaging, DAPI staining, and residual DNA quantification. After decellularization, most visible cellular structures were removed, and DAPI-positive nuclei were absent, indicating effective removal of cellular nuclear components (Supplementary Figure S1A). Consistently, the residual DNA content in HE-ECM was approximately 16.28 ng/mg dry ECM, which was markedly below the commonly used threshold of 50 ng/mg dry ECM for decellularized ECM (Crapo et al., 2011) (Supplementary Figure S1B). These results support the effective decellularization of HE-ECM while preserving residual matrix material for subsequent characterization.

The biochemical composition of HE-ECM was then assessed by Coomassie brilliant blue staining, hydroxyproline-based collagen quantification, and total carbohydrate assay. Coomassie brilliant blue staining showed a detectable protein band in HE-ECM within a molecular weight range similar to bovine collagen, supporting the presence of collagen-associated protein components. Hydroxyproline and total carbohydrate assays were performed to quantify key biochemical components of HE-ECM (Supplementary Figure S1C). Both standard curves showed good linearity over the tested concentration ranges, with regression equations of y = 0.04295x for hydroxyproline (R^2^ = 0.99905) and y = 0.01615x for D-glucose (R^2^ = 0.99941) (Supplementary Figures S1D, F). Based on hydroxyproline quantification and a hydroxyproline-to-collagen conversion factor of 7.46, the collagen content of HE-ECM was calculated to be 24.3% (Supplementary Figure S1E). The total carbohydrate content, expressed as D-glucose equivalents, was 17.4 mg/g, corresponding to 1.74% of sample weight (Supplementary Figure S1G). Together, these results support effective decellularization and confirm that HE-ECM contains collagen-rich and carbohydrate-containing matrix components.

### 3.2 Physicochemical characterization of HE-ECM

To evaluate the physicochemical properties of recombinant collagen and HE-ECM, a series of morphological, structural, and thermal analyses were performed.

**FIGURE 2:**
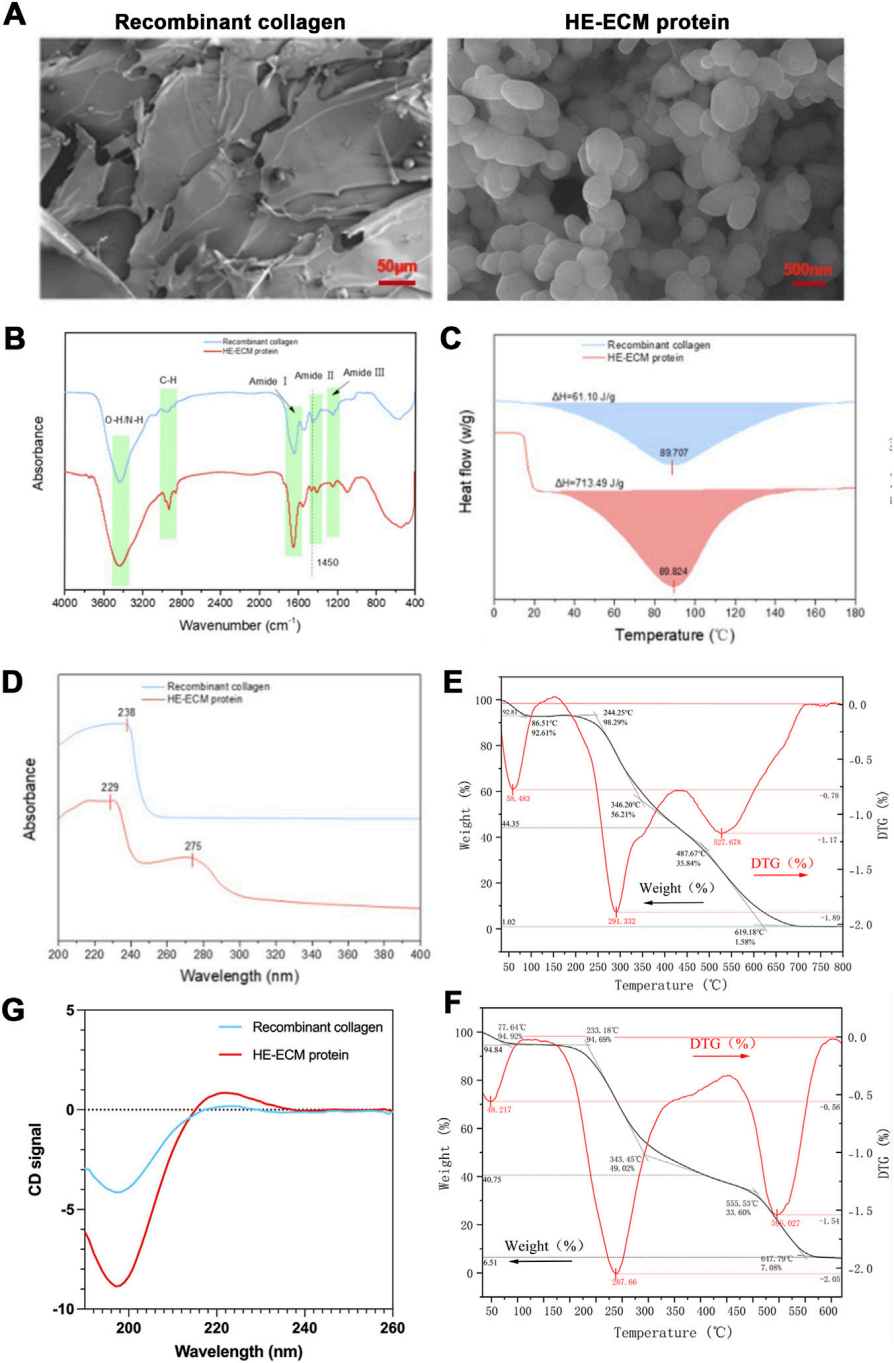
Characterization of HE-ECM. **(A)** Representative SEM images showing the surface morphology of recombinant collagen (left) and HE-ECM (right). Scale bar, 50 μm and 500 nm, respectively. **(B)** FTIR spectra of recombinant collagen and HE-ECM showing the characteristic amide I, II, and III bands. **(C)** DSC thermograms of recombinant collagen and HE-ECM. **(D)** UV–Vis absorption spectra of recombinant collagen and HE-ECM. **(E)** TGA and DTG curves of recombinant collagen. **(F)** TGA and DTG curves of HE-ECM. **(G)** CD spectra of recombinant collagen and HE-ECM recorded.

SEM analysis revealed distinct morphological differences between recombinant collagen and HE-ECM. Recombinant collagen exhibited a sheet-like, relatively compact surface, whereas HE-ECM presented a porous network resembling ECM, which is considered more favorable for cellular infiltration and nutrient exchange (Figure 2A).

FTIR spectra of both groups displayed characteristic collagen peaks, including amide I (∼1,650 cm^−1^), amide II (∼1,550 cm^−1^), and amide III (∼1,240 cm^−1^) (Figure 2B). Notably, the amide III band, which is a collagen-specific absorption peak reflecting intramolecular hydrogen-bonding arrangement and stability, together with the 1,450 cm^−1^ band, which is associated with the overall protein structure, provides a sensitive indicator of triple-helix integrity. In HE-ECM, the intensity ratio of amide III to the 1,450 cm^−1^ band was approximately 1.0, whereas the ratio decreased in recombinant collagen, which suggested that the triple-helical conformation of collagen was well preserved with superior structural stability in HE-ECM.

DSC analysis demonstrated that HE-ECM had a higher denaturation temperature (T_m_) and enthalpy change (ΔH) compared with recombinant collagen, suggesting improved thermal stability and stronger intermolecular interactions (Figure 2C).

In the results of UV–Vis spectroscopy, recombinant collagen exhibited a single strong absorption peak at 238 nm, corresponding to the peptide bond signal of collagen (Figure 2D). In contrast, HE-ECM displayed two distinct peaks at 229 nm and 275 nm. The peak at 229 nm reflects peptide bond absorption, while the additional peak at 275 nm corresponds to aromatic amino acid residues, indicating that HE-ECM retains a more complex and native-like protein composition than recombinant collagen.

TGA revealed that HE-ECM had a higher onset decomposition temperature and a greater residual mass fraction after high-temperature degradation compared with recombinant collagen, suggesting improved thermal resistance and a greater proportion of thermally stable matrix-associated components (Figures 2E,F). The DTG curves showed multistage degradation behavior in both materials, corresponding to moisture loss and thermal decomposition of proteinaceous components. Overall, HE-ECM displayed a more gradual mass-loss profile than recombinant collagen, which may be associated with its more complex ECM composition and preserved intermolecular interactions.

CD spectroscopy was performed to further evaluate the secondary structural features of recombinant collagen and HE-ECM (Figure 2G). Both samples showed characteristic collagen-related CD profiles, with a negative band near 197–198 nm and a positive band near 221–223 nm. Compared with recombinant collagen, HE-ECM exhibited a stronger negative signal around 197 nm and a more pronounced positive signal near 222 nm. These results support the presence of collagen-like triple-helical structural features in HE-ECM.

Collectively, these findings demonstrate that HE-ECM maintains a more native-like structure, superior stability, and a more complex protein composition with preserved ECM components than recombinant collagen, highlighting its potential as a biomimetic scaffold.

### 3.3 HE-ECM exhibited enhanced abilities to promote cell proliferation and migration *in vitro*

To assess the cytocompatibility and bioactivity of recombinant collagen and HE-ECM, the viability, proliferation, and migration of L929 fibroblasts were evaluated using Live/Dead staining, CCK-8 assays, and scratch wound assays.

**FIGURE 3:**
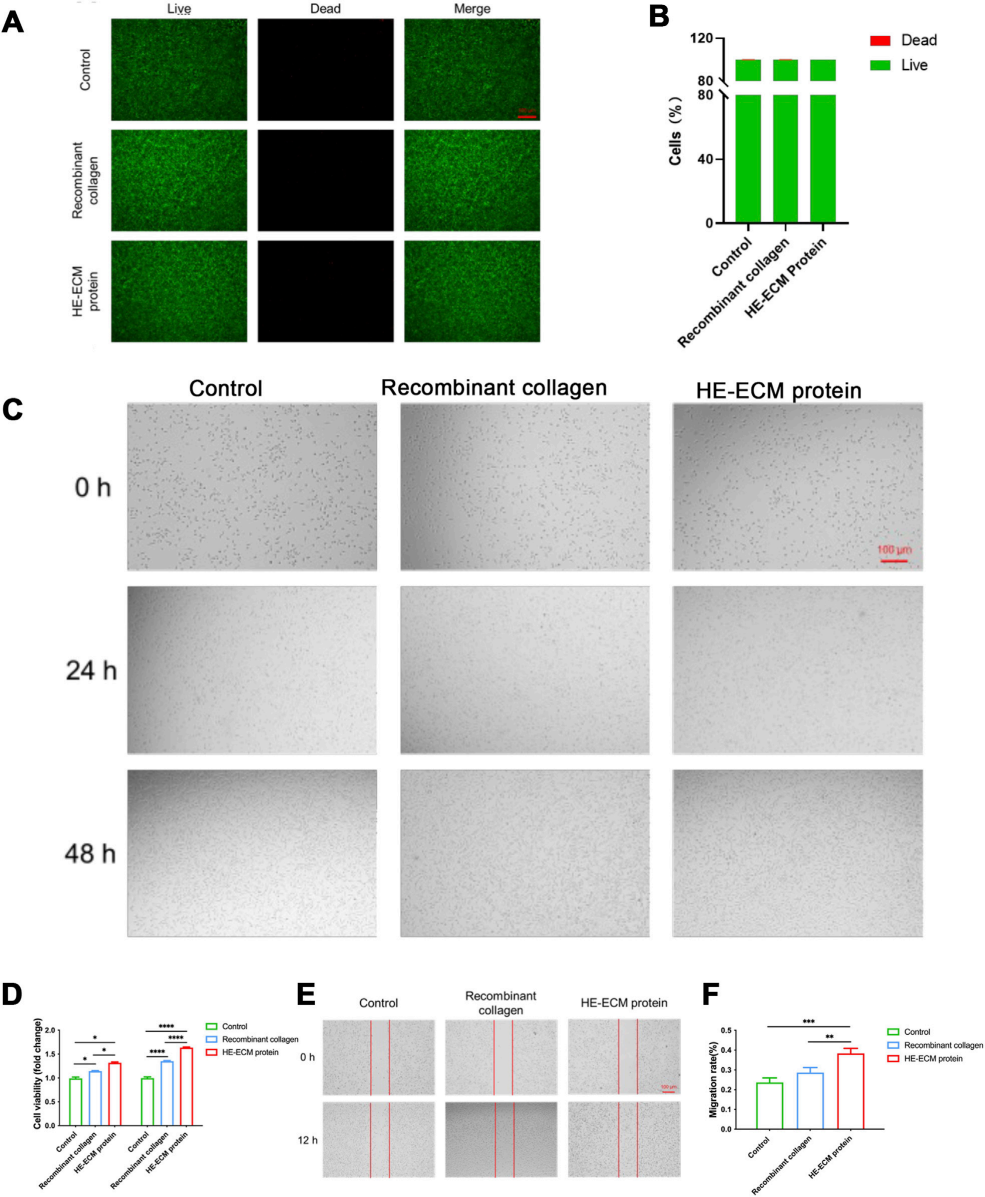
Cellular response of L929 fibroblasts cultured with recombinant collagen and HE-ECM. **(A,B)** Representative Live/Dead staining images of fibroblasts and the quantification data after 24 h of co-culture. Live cells stained with Calcein-AM (green) and dead cells with PI (red) (Scale bar: 500 μm) (n = 3). **(C,D)** CCK-8 assay of fibroblast proliferation and the quantification of cell viability at 24 h and 48 h in different treatment groups (Scale bar, 100 μm) (n = 3). **(E,F)** Scratch assay images of fibroblasts at 0 h and 12 h and the quantification of migration rate at 12 h in different treatment groups (Scale bar: 100 μm) (n = 3) (*P < 0.05; **P < 0.01; ***P < 0.001; and ****P < 0.0001).

Live/Dead staining revealed that most fibroblasts remained viable after 24 h of co-culture in all groups, with cells exhibiting predominantly green fluorescence and minimal red signals (Figure 3A). Quantitative analysis showed no evidence of cytotoxicity for either recombinant collagen or HE-ECM, while the HE-ECM group maintained a slightly higher percentage of live cells compared with recombinant collagen and the control group (Figure 3B). CCK-8 assays further demonstrated that fibroblasts proliferated in a time-dependent manner across all groups (Figure 3C). At both 24 h and 48 h, cell viability in the HE-ECM group was significantly higher than that in the recombinant collagen and control groups, indicating that HE-ECM provided a more favorable microenvironment for fibroblast growth (Figure 3D).

Migration assay results demonstrated higher migration rates in the HE-ECM group than those in the recombinant collagen and control groups at 12 h, indicating an enhanced migratory capability of fibroblasts cultured with HE-ECM (Figures 3E,F).

Together, these *in vitro* experiments demonstrated that both recombinant collagen and HE-ECM were cytocompatible, and HE-ECM more effectively promoted fibroblast viability, proliferation, and migration, highlighting its superior bioactivity for wound healing applications.

### 3.4 HE-ECM accelerates wound closure *in vivo*

**FIGURE 4:**
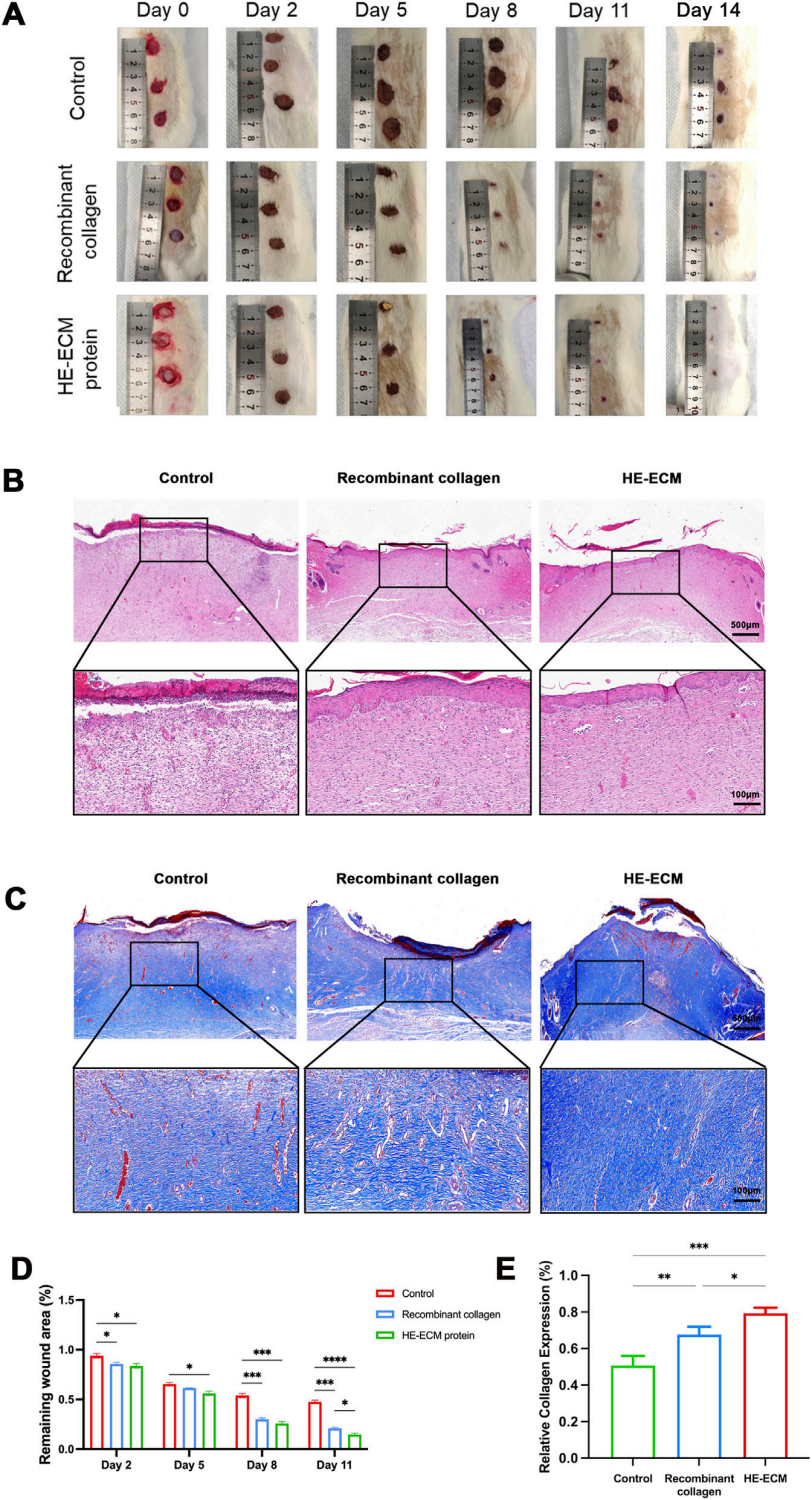
*In vivo* wound healing in a rat full-thickness skin defect model. **(A)** Representative macroscopic images of wounds treated with saline, recombinant collagen, or HE-ECM at days 0, 2, 5, 8, 11, and 14 (n = 6). **(B)** Representative H&E staining images of wound sections at day 14 (Scale bar: 500 μm and 100 μm). **(C)** Representative Masson’s trichrome staining images showing collagen deposition at day 14 (Scale bar: 500 μm and 100 μm). **(D)** Quantification of wound closure rates in the indicated groups. **(E)** Quantitative analysis of collagen deposition by Masson trichrome staining (*P < 0.05; **P < 0.01; ***P < 0.001; and ****P < 0.0001).

The *in vivo* wound healing performance of recombinant collagen and HE-ECM was further evaluated using a rat full-thickness skin defect model. Macroscopic observation showed progressive wound closure in all groups over a 14-day period (Figure 4A). Notably, wounds treated with HE-ECM exhibited more rapid contraction and were nearly closed by day 11, whereas recombinant collagen-treated wounds showed relatively slower healing, and wounds in the control group remained largely unhealed. Consistently, quantitative analysis demonstrated that the wound closure rate in the HE-ECM group was significantly higher than that in the recombinant collagen and control groups at both day 8 and day 11 (Figure 4D).

Histological analyses further supported the superior wound healing capacity of HE-ECM. H&E staining revealed that, compared with the control group, both recombinant collagen- and HE-ECM–treated wounds exhibited more organized granulation tissue formation and improved re-epithelialization (Figure 4B). In addition, pronounced inflammatory cell infiltration was observed in the control group, whereas inflammatory infiltration was markedly reduced in the recombinant collagen and HE-ECM groups, suggesting that HE-ECM treatment may be associated with a moderated inflammatory response during wound healing. Masson’s trichrome staining further demonstrated enhanced ECM remodeling in the HE-ECM group. Wounds treated with HE-ECM displayed denser and more orderly collagen deposition, resembling the architecture of normal skin, whereas collagen fibers in the recombinant collagen and control groups appeared relatively sparse or disorganized (Figure 4C). Quantitative analysis confirmed that collagen deposition was significantly increased in the material-treated groups compared with the control group, with higher collagen content observed in the HE-ECM group than in the recombinant collagen group (Figure 4E).

Collectively, these *in vivo* results indicate that HE-ECM promotes faster wound closure, supports organized tissue regeneration, and enhances collagen matrix formation compared with recombinant collagen.

### 3.5 Proteomic profiling and functional analysis of HE-ECM

**FIGURE 5:**
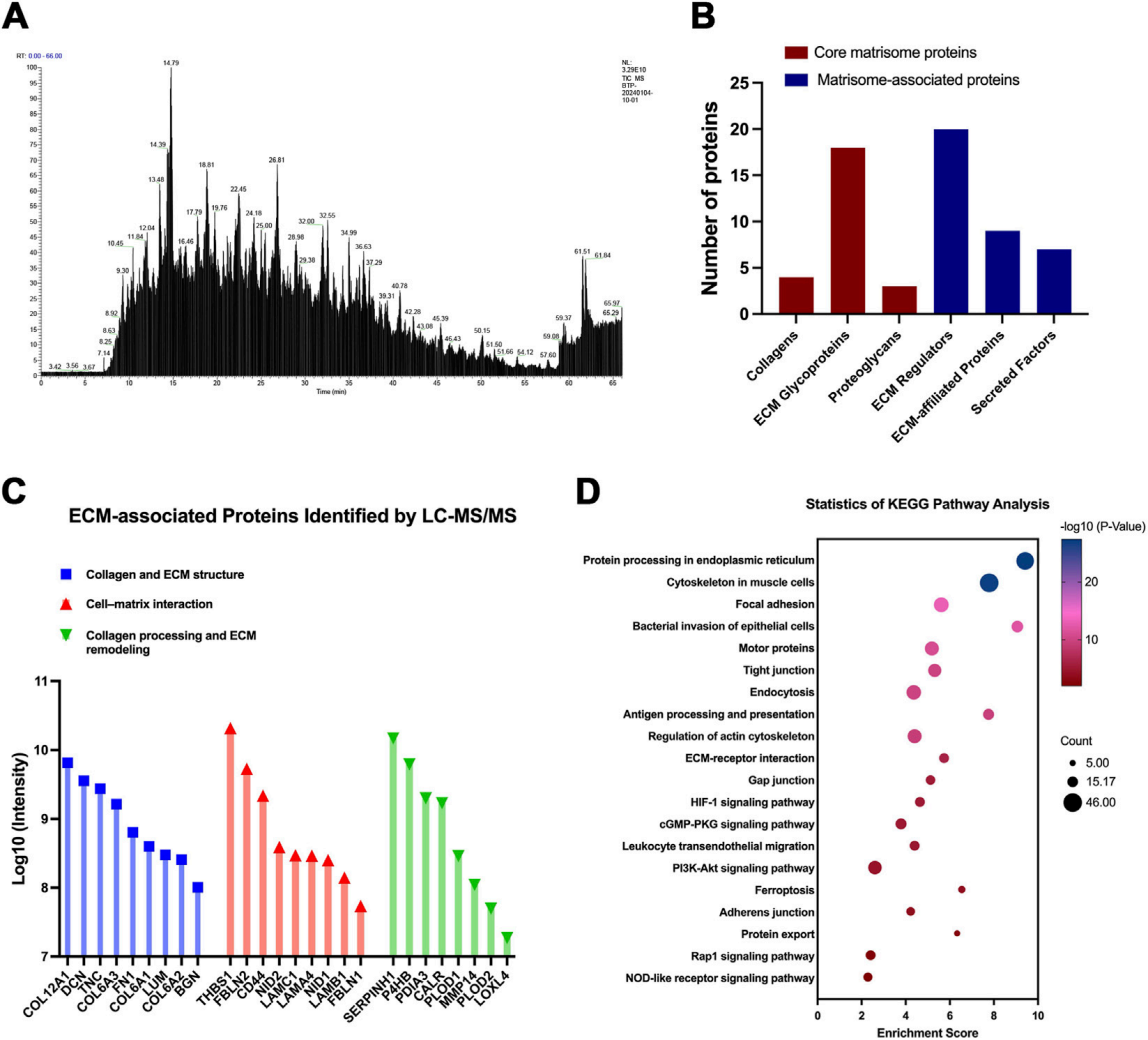
Proteomic profiling and functional analysis of HE-ECM. **(A)** Distribution of LC–MS/MS features across the chromatographic gradient. **(B)** Matrisome category distribution of identified HE-ECM proteins. **(C)** Functional classification of representative ECM-associated proteins identified in HE-ECM by LC–MS/MS. Proteins were grouped into collagen and ECM structure, cell–matrix interaction, and collagen processing and ECM remodeling, and their relative abundance is presented as log10-transformed intensity. **(D)** KEGG pathway enrichment analysis of the identified proteins, highlighting pathways related to cell–matrix interactions, cytoskeletal regulation, and signaling processes relevant to tissue repair.

Proteomic analysis was performed as an exploratory compositional profiling of HE-ECM. Peptide ion signals were detected throughout the entire retention time window (0–66 min), indicating stable chromatographic separation and reliable mass spectrometric acquisition (Figure 5A). Matrisome annotation further classified 61 proteins as matrisome or matrisome-associated proteins, including 25 core matrisome proteins and 36 matrisome-associated proteins. Specifically, the identified matrisome proteins included 4 collagens, 18 ECM glycoproteins, 3 proteoglycans, 20 ECM regulators, 9 ECM-affiliated proteins, and 7 secreted factors (Figure 5B). Representative ECM-associated proteins identified by LC–MS/MS were further classified according to their functional relevance (Figure 5C). Representative ECM-related proteins identified in HE-ECM were grouped into three functional categories. Structural ECM components included collagens and matrix-associated proteins such as COL12A1, COL6A1, COL6A2, COL6A3, DCN, LUM, BGN, FN1, and TNC. Cell-matrix interaction-related proteins included THBS1, CD44, LAMA4, LAMB1, LAMC1, NID1, NID2, FBLN1, and FBLN2. Proteins associated with collagen processing and ECM remodeling included SERPINH1, P4HB, PDIA3, CALR, PLOD1, PLOD2, MMP14, and LOXL4. These results support the presence of a complex ECM-associated protein composition in HE-ECM, including structural, adhesive, and remodeling-related matrix components. Functional enrichment analysis was performed using the identified HE-ECM-associated proteins to explore their related biological annotations. The enriched terms included protein processing in the endoplasmic reticulum, regulation of actin cytoskeleton, focal adhesion, and ECM–receptor interaction (Figure 5D). The enrichment of protein processing in the endoplasmic reticulum is consistent with the presence of collagen chaperones and folding-related proteins, such as SERPINH1/HSP47, P4HB, PDIA3, and CALR, which may reflect the collagen-producing fibroblast origin of HE-ECM and the retention of collagen-associated biosynthetic components in the cell-derived matrix. In addition, enrichment of focal adhesion, ECM–receptor interaction, regulation of actin cytoskeleton, and motor protein pathways was consistent with the presence of ECM and matrix-associated proteins involved in cell-matrix adhesion, mechanotransduction, cytoskeletal organization, and migration-related processes. Pathways associated with tissue repair and microenvironmental regulation, including PI3K–Akt, HIF-1, Rap1, and endocytosis signaling, were also represented, further providing exploratory clues regarding potential biological processes.

## 4 Discussion

Skin wound healing remains a significant clinical challenge. Despite advances in wound management strategies, achieving rapid closure while restoring functional tissue architecture remains difficult (Sen et al., 2009; Rowan et al., 2015). A central unmet need lies in the development of biomaterials that can faithfully reproduce the human extracellular matrix (ECM) microenvironment, particularly its collagen-rich architecture, which is essential for coordinated cellular migration, matrix remodeling, and tissue restoration. In this study, we developed an engineered and scalable strategy to fabricate HE-ECM and systematically evaluated its physicochemical properties and biological performance in comparison with recombinant collagen. Our results demonstrate that HE-ECM exhibits enhanced biomimetic characteristics and promotes wound healing both *in vitro* and *in vivo*. Notably, the innovation of this work lies in establishing a controllable and cell-line-based production approach for HE-ECM, thereby addressing the long-standing limitations of ECM availability, while simultaneously developing a new biomaterial that demonstrates advantages over recombinant collagen in promoting skin repair.

To generate biomaterials that can be produced at scale while faithfully recapitulating the native tissue microenvironment, extensive efforts have been devoted to ECM-based strategies (Xu et al., 2024; Suhaeri et al., 2018; Lin et al., 2012; Bourget et al., 2012). ECM is a highly complex and dynamic network composed of collagens, fibronectin, laminins, glycosaminoglycans, and other bioactive molecules, among which collagen serves as the predominant structural component (Aamodt and Grainger, 2016). ECM is synthesized by resident cells and, in turn, actively regulates cellular behavior by providing structural support and activating multiple signaling pathways essential for cell adhesion, migration, proliferation, and differentiation (Rosso et al., 2004). Therefore, reproducing the composition and architecture of native ECM, particularly its collagen-dominated framework, is critical for accelerating wound healing and restoring normal skin structure and function.

Current strategies to obtain human-relevant collagen primarily follow two distinct routes: recombinant collagen produced through genetic engineering and ECM generated directly by human cells. Recombinant collagen currently represents the dominant approach for generating human collagen materials and was originally developed to approximate human collagen while avoiding the immunogenicity and biosafety concerns associated with animal-derived sources. However, despite its advantages in purity and sequence control, recombinant collagen remains a simplified representation of native human collagen (Guo et al., 2024; Cheng et al., 2024). Limitations in post-translational modification, molecular assembly, and fibrillar organization result in reduced structural stability, accelerated degradation, and incomplete biological signaling, which together constrain its regenerative performance in wound healing applications (Cao et al., 2024; He et al., 2025; O'Donnell et al., 2019). These intrinsic constraints highlight the need for alternative approaches capable of generating collagen within a more physiologically relevant human ECM context. In this study, we addressed this challenge by developing an engineered and scalable strategy to fabricate HE-ECM using cultured human fibroblasts as a direct source of human collagen and associated ECM components. Compared with recombinant collagen, our HE-ECM retained a more complex protein composition with preserved ECM components, maintained collagen triple-helical integrity and exhibited better structural stability. These properties translated into significantly enhanced fibroblast proliferation and migration *in vitro*, as well as accelerated wound closure and improved tissue organization *in vivo*.

The engineered fabrication strategy of HE-ECM suggests potential advantages in manufacturing efficiency and scalability. In this study, scalability refers specifically to an optimized and standardized production strategy based on commercially available HFFs and the parallel expansion of identical culture units. Cell-derived ECM is commonly produced by culturing cells for extended periods in various formats, including two-dimensional monolayers, scaffold coatings, three-dimensional multicellular aggregates, or biodegradable carriers (Fitzpatrick and McDevitt, 2015; Dzobo et al., 2019). In our study, HE-ECM was generated using a two-dimensional fibroblast monolayer culture system with biochemical induction, enabling controlled ECM deposition and efficient scalability. This workflow yielded approximately 125 g of wet HE-ECM pellet per batch before lyophilization. Mechanical stimulation or modulation of environmental conditions can be further applied to enhance ECM deposition and improve biomimicry (Syedain et al., 2011; Pei et al., 2013). Once sufficient ECM has accumulated, cellular components are removed through combinations of chemical, physical, and/or enzymatic decellularization methods, yielding a biologically active matrix with preserved structural and biochemical cues. Compared with decellularized ECM extracted directly from tissues or organs (Dzobo et al., 2019; Choudhury et al., 2018; Gilbert et al., 2006), cell-derived ECM offers greater flexibility for *in vitro* manipulation, characterization, and standardization, and is more amenable to scalable and engineered production (Fitzpatrick and McDevitt, 2015; Dzobo et al., 2019). It allows better control over the cell source, culture conditions, and batch processing, while reducing dependence on animal tissues and species-related variability.

Fibroblasts are the predominant cell type in connective tissues and are particularly effective in generating collagen-rich ECM (Fitzpatrick and McDevitt, 2015). In this study, HE-ECM was produced from a single commercial HFF source, which allowed standardized culture and processing conditions and enabled evaluation of batch-to-batch consistency. These advantages make HFF-derived ECM an attractive strategy for developing reproducible and engineered biomimetic biomaterials for wound repair and regenerative medicine. Consistent with commercial products of ECM materials such as Biobrane, Integra, AlloDerm and Graftskin (Dzobo et al., 2019; Choudhury et al., 2018; Winfrey et al., 1999; Saffle, 2009), which validate the therapeutic potential of ECM-based wound repair, the HE-ECM developed in this study uniquely represents a fully human, cell-derived ECM produced through a reproducible and engineered fabrication strategy. However, ECM generated from different donor-derived fibroblast lines may still vary in composition and biological activity. Future studies will therefore compare multiple donor-derived HFF sources and establish release criteria to control donor-related variability.

HE-ECM is a complex cell-derived matrix, whereas recombinant collagen is a purified single-component material. Therefore, the improved performance of HE-ECM should be interpreted as the effect of a multi-component matrix microenvironment rather than collagen alone. This distinction is particularly relevant because wound healing requires coordinated regulation of cell adhesion, migration, proliferation, angiogenesis, inflammation, and matrix remodeling, which are difficult to support fully with a single purified component (Peña and Martin, 2024). The proteomic analysis revealed that HE-ECM contains a broad spectrum of matrisome and matrix-associated proteins, including collagens, ECM glycoproteins, proteoglycans, ECM regulators, and secreted factors. Exploratory pathway annotation further indicated associations with collagen processing, cell-matrix interaction, focal adhesion, and cytoskeletal regulation. SERPINH1/HSP47, P4HB, PDIA3, and CALR are involved in collagen folding, post-translational processing, secretion, and ECM deposition, which is consistent with the fibroblast-derived and collagen-rich nature of HE-ECM (Diller and Tabor, 2022; Rousselle et al., 2019; Bunel et al., 2024; Li et al., 2016). In addition, fibronectin, a representative ECM glycoprotein detected in HE-ECM, participates in matrix assembly through fibronectin–integrin interactions and can link ECM ligands to cytoskeletal organization through integrin-mediated adhesion complexes (Kanchanawong and Calderwood, 2023; Mao and Schwarzbauer, 2005). Furthermore, histological analyses demonstrated more organized collagen deposition in HE-ECM-treated wounds, suggesting that HE-ECM may facilitate orderly ECM remodeling during wound repair. Together, these findings suggest that HE-ECM may provide broader structural and bioactive cues than collagen alone. Although detailed mechanistic pathways were not fully elucidated in the present study, the observed enrichment of signaling pathways such as PI3K-Akt and HIF-1 suggests potential involvement in cell survival, stress adaptation, and vascular responses within the wound microenvironment (Hu et al., 2021; Chen et al., 2021; Huang et al., 2025; Jung et al., 2025; Pang et al., 2021). These coordinated effects may be associated with the enhanced regenerative performance of HE-ECM compared with recombinant collagen. Future studies involving targeted pathway inhibition, immune profiling, angiogenesis markers, and re-epithelialization analysis will be needed to define the specific mechanisms underlying the regenerative effects of HE-ECM and the individual contribution of specific matrix components.

Despite its promising performance, this study has several limitations. Although proteomic analysis provided insights into the functional composition of HE-ECM, the quantitative contribution of collagen to tissue regeneration, as well as the detailed molecular mechanisms underlying its pro-regenerative effects, were not systematically investigated. Future studies will focus on dissecting the specific role of collagen within HE-ECM, for example, by selectively replacing endogenous human collagen with recombinant collagen to more precisely evaluate collagen-dependent *versus* matrix-context–dependent effects. In addition, mechanistic investigations incorporating targeted pathway inhibition, transcriptomic analysis, or immune profiling will be necessary to elucidate the precise signaling mechanisms involved. Moreover, future studies should evaluate HE-ECM generated from multiple donor-derived HFF lines and other relevant human cell sources, include validation based on human dermal fibroblasts to further evaluate the human-specific biological effects, and optimize mechanical stimulation and culture conditions to improve ECM reproducibility, deposition, and biomimicry. Mechanical properties, including rheological behavior, compressive/tensile modulus, and quantitative pore or fibril metrics, should also be included in future material optimization. Finally, long-term *in vivo* studies and evaluations in disease-specific wound models, such as diabetic or ischemic wounds, are warranted to further assess the translational potential of HE-ECM.

## 5 Conclusion

In this study, we established an engineered and scalable strategy to fabricate HE-ECM and systematically compared its physicochemical properties and regenerative performance with recombinant collagen. Our results demonstrated that HE-ECM more closely recapitulated native ECM architecture and composition, with human collagen embedded in its native matrix context, thereby exhibiting improved bioactivity in promoting cell proliferation, migration, and wound healing *in vivo*. The innovation of this work lies in the reproducible, *in vitro* production of HE-ECM that reduces direct dependence on human tissue sources and addresses the limited availability of human collagen sources, together with a comprehensive comparison against recombinant collagen across multiple evaluation levels, providing evidence supporting HE-ECM as a more biomimetic alternative to recombinant collagen. Future studies will focus on further standardization, scale-up manufacturing, and integration of HE-ECM with advanced delivery systems to facilitate its clinical translation in wound healing and regenerative medicine.

## Data Availability

The original contributions presented in the study are included in the article/Supplementary Material, further inquiries can be directed to the corresponding authors.

## Supplementary material

SUPPLEMENTARY FIGURE S1 Biochemical validation of HE-ECM. **(A)** Bright-field images before and after decellularization (above). DAPI staining images before and after decellularization (below) (Scale bar, 50 μm and 200 μm, respectively). **(B)** Quantification of residual DNA content in HE-ECM. The dashed line indicates the commonly accepted threshold for decellularized ECM, 50 ng DNA/mg dry ECM (Crapo et al., 2011). **(C)** Coomassie brilliant blue staining of SDS-PAGE gel showing the protein profiles of HE-ECM and bovine type I collagen. **(D)** The L-hydroxyproline standard curve. **(E)** Hydroxyproline-based quantification of collagen content in HE-ECM. **(F)** The D-glucose standard curve. **(G)** Total carbohydrate content in HE-ECM.

## References

[B1] Aamodt J. M. Grainger D. W. (2016). Extracellular matrix-based biomaterial scaffolds and the host response. Biomaterials 86, 68–82. 10.1016/j.biomaterials.2016.02.003 26890039 PMC4785021

[B2] Ahlfors J. E. Billiar K. L. (2007). Biomechanical and biochemical characteristics of a human fibroblast-produced and remodeled matrix. Biomaterials 28, 2183–2191. 10.1016/j.biomaterials.2006.12.030 17280714

[B3] Assunção M. Dehghan-Baniani D. Yiu C. H. K. Später T. Beyer S. Blocki A. (2020). Cell-derived extracellular matrix for tissue engineering and regenerative medicine. Front. Bioeng. Biotechnol. 8, 602009. 10.3389/fbioe.2020.602009 33344434 PMC7744374

[B4] Avila Rodríguez M. I. Rodríguez Barroso L. G. Sánchez M. L. (2018). Collagen: a review on its sources and potential cosmetic applications. J. Cosmet. Dermatol. 17, 20–26. 10.1111/jocd.12450 29144022

[B5] Bourget J. M. Gauvin R. Larouche D. Lavoie A. Labbé R. Auger F. A. (2012). Human fibroblast-derived ECM as a scaffold for vascular tissue engineering. Biomaterials 33, 9205–9213. 10.1016/j.biomaterials.2012.09.015 23031531

[B6] Bunel L. Pincet L. Malhotra V. Raote I. Pincet F. (2024). A model for collagen secretion by intercompartmental continuities. Proc. Natl. Acad. Sci. U. S. A. 121, e2310404120. 10.1073/pnas.2310404120 38147551 PMC10769856

[B7] Cao L. Zhang Z. Yuan D. Yu M. Min J. (2024). Tissue engineering applications of recombinant human collagen: a review of recent progress. Front. Bioeng. Biotechnol. 12, 1358246. 10.3389/fbioe.2024.1358246 38419725 PMC10900516

[B8] Chen J. Huang Y. Hu X. Bian X. Nian S. (2021). Gastrodin prevents homocysteine-induced human umbilical vein endothelial cells injury via PI3K/Akt/eNOS and Nrf2/ARE pathway. J. Cell. Mol. Med. 25, 345–357. 10.1111/jcmm.16073 33320446 PMC7810955

[B9] Cheng Z. Hong B. Li Y. Wang J. (2024). Preparation and characterization of hydroxylated recombinant collagen by incorporating proline and hydroxyproline in proline-deficient *Escherichia coli* . Bioeng. (Basel) 11, 975. 10.3390/bioengineering11100975 PMC11504287 39451351

[B10] Choudhury D. Tun H. W. Wang T. Naing M. W. (2018). Organ-derived decellularized extracellular matrix: a game changer for bioink manufacturing? Trends Biotechnol. 36, 787–805. 10.1016/j.tibtech.2018.03.003 29678431

[B11] Crapo P. M. Gilbert T. W. Badylak S. F. (2011). An overview of tissue and whole organ decellularization processes. Biomaterials 32, 3233–3243. 10.1016/j.biomaterials.2011.01.057 21296410 PMC3084613

[B12] Davison-Kotler E. Marshall W. S. García-Gareta E. (2019). Sources of collagen for biomaterials in skin wound healing. Bioeng. (Basel) 6, 56. 10.3390/bioengineering6030056 31261996 PMC6783949

[B13] Diller R. B. Tabor A. J. (2022). The role of the extracellular matrix (ECM) in wound healing: a review. Biomimetics (Basel) 7, 87. 10.3390/biomimetics7030087 35892357 PMC9326521

[B14] Dzobo K. Motaung K. Adesida A. (2019). Recent trends in decellularized extracellular matrix bioinks for 3D printing: an updated review. Int. J. Mol. Sci. 20, 4628. 10.3390/ijms20184628 31540457 PMC6788195

[B15] Eriksson E. Liu P. Y. Schultz G. S. Martins-Green M. Tanaka R. Weir D. (2022). Chronic wounds: treatment consensus. Wound Repair Regen. 30, 156–171. 10.1111/wrr.12994 35130362 PMC9305950

[B16] Fitzpatrick L. E. McDevitt T. C. (2015). Cell-derived matrices for tissue engineering and regenerative medicine applications. Biomater. Sci. 3, 12–24. 10.1039/c4bm00246f 25530850 PMC4270054

[B17] Gilbert T. W. Sellaro T. L. Badylak S. F. (2006). Decellularization of tissues and organs. Biomaterials 27, 3675–3683. 10.1016/j.biomaterials.2006.02.014 16519932

[B18] Guo X. Ma Y. Wang H. Yin H. Shi X. Chen Y. (2024). Status and developmental trends in recombinant collagen preparation technology. Regen. Biomater. 11, rbad106. 10.1093/rb/rbad106 38173768 PMC10761200

[B19] He H. Ye M. Cui G. Xiao J. (2025). Recombinant collagen in regenerative medicine: expression strategies, structural design, and translational applications. Mater. Today Bio 35, 102452. 10.1016/j.mtbio.2025.102452 41293490 PMC12642170

[B20] Hu Y. Tao R. Chen L. Xiong Y. Xue H. Hu L. (2021). Exosomes derived from pioglitazone-pretreated MSCs accelerate diabetic wound healing through enhancing angiogenesis. J. Nanobiotechnol. 19, 150. 10.1186/s12951-021-00894-5 34020670 PMC8139165

[B21] Huang J. Deng Q. Tsang L. L. Chang G. Guo J. Ruan Y. C. (2025). Mesenchymal stem cells from perinatal tissues promote diabetic wound healing via PI3K/AKT activation. Stem Cell Res. Ther. 16, 59. 10.1186/s13287-025-04141-8 39923118 PMC11807333

[B22] Jung J. P. Olutoye O. O. 2nd Prajapati T. J. Jung O. S. Yutzy L. D. Nguyen K. L. (2025). Sustained ROS scavenging and pericellular oxygenation by lignin composites rescue HIF-1α and VEGF levels to improve diabetic wound neovascularization and healing. Acta Biomater. 202, 205–215. 10.1016/j.actbio.2025.04.047 40286890 PMC13361895

[B23] Kanchanawong P. Calderwood D. A. (2023). Organization, dynamics and mechanoregulation of integrin-mediated cell-ECM adhesions. Nat. Rev. Mol. Cell Biol. 24, 142–161. 10.1038/s41580-022-00531-5 36168065 PMC9892292

[B24] Li Q. Uygun B. E. Geerts S. Ozer S. Scalf M. Gilpin S. E. (2016). Proteomic analysis of naturally-sourced biological scaffolds. Biomaterials 75, 37–46. 10.1016/j.biomaterials.2015.10.011 26476196 PMC4644483

[B25] Lin H. Yang G. Tan J. Tuan R. S. (2012). Influence of decellularized matrix derived from human mesenchymal stem cells on their proliferation, migration and multi-lineage differentiation potential. Biomaterials 33, 4480–4489. 10.1016/j.biomaterials.2012.03.012 22459197

[B26] Mao Y. Schwarzbauer J. E. (2005). Fibronectin fibrillogenesis, a cell-mediated matrix assembly process. Matrix Biol. 24, 389–399. 10.1016/j.matbio.2005.06.008 16061370

[B27] Naomi R. Ridzuan P. M. Bahari H. (2021). Current insights into collagen type I. Polym. (Basel) 13, 2642. 10.3390/polym13162642 PMC8399689 34451183

[B28] O'Donnell B. T. Ives C. J. Mohiuddin O. A. Bunnell B. A. (2019). Beyond the present constraints that prevent a wide spread of tissue engineering and regenerative medicine approaches. Front. Bioeng. Biotechnol. 7, 95. 10.3389/fbioe.2019.00095 31134194 PMC6514054

[B29] Pang L. Tian P. Cui X. Wu X. Zhao X. Wang H. (2021). *In situ* photo-cross-linking hydrogel accelerates diabetic wound healing through restored hypoxia-inducible factor 1-alpha pathway and regulated inflammation. ACS Appl. Mater. Interfaces 13, 29363–29379. 10.1021/acsami.1c07103 34128630

[B30] Pei M. Zhang Y. Li J. Chen D. (2013). Antioxidation of decellularized stem cell matrix promotes human synovium-derived stem cell-based chondrogenesis. Stem Cells Dev. 22, 889–900. 10.1089/scd.2012.0495 23092115 PMC3585742

[B31] Peña O. A. Martin P. (2024). Cellular and molecular mechanisms of skin wound healing. Nat. Rev. Mol. Cell Biol. 25, 599–616. 10.1038/s41580-024-00715-1 38528155

[B32] Petrov P. B. Considine J. M. Izzi V. Naba A. (2023). Matrisome AnalyzeR - a suite of tools to annotate and quantify ECM molecules in big datasets across organisms. J. Cell Sci. 136, jcs261255. 10.1242/jcs.261255 37555624 PMC10499032

[B33] Rosso F. Giordano A. Barbarisi M. Barbarisi A. (2004). From cell-ECM interactions to tissue engineering. J. Cell. Physiol. 199, 174–180. 10.1002/jcp.10471 15039999

[B34] Rousselle P. Montmasson M. Garnier C. (2019). Extracellular matrix contribution to skin wound re-epithelialization. Matrix Biol. 75–76, 12–26. 10.1016/j.matbio.2018.01.002 29330022

[B35] Rowan M. P. Cancio L. C. Elster E. A. Burmeister D. M. Rose L. F. Natesan S. (2015). Burn wound healing and treatment: review and advancements. Crit. Care 19, 243. 10.1186/s13054-015-0961-2 26067660 PMC4464872

[B36] Saffle J. R. (2009). Closure of the excised burn wound: temporary skin substitutes. Clin. Plast. Surg. 36, 627–641. 10.1016/j.cps.2009.05.005 19793557

[B37] Sen C. K. Gordillo G. M. Roy S. Kirsner R. Lambert L. Hunt T. K. (2009). Human skin wounds: a major and snowballing threat to public health and the economy. Wound Repair Regen. 17, 763–771. 10.1111/j.1524-475X.2009.00543.x 19903300 PMC2810192

[B38] Sionkowska A. Adamiak K. Musiał K. Gadomska M. (2020). Collagen based materials in cosmetic applications: a review. Mater. (Basel) 13, 4217. 10.3390/ma13194217 32977407 PMC7578929

[B39] Suhaeri M. Noh M. H. Moon J. H. Kim I. G. Oh S. J. Ha S. S. (2018). Novel skin patch combining human fibroblast-derived matrix and ciprofloxacin for infected wound healing. Theranostics 8, 5025–5038. 10.7150/thno.26837 30429884 PMC6217057

[B40] Syedain Z. H. Meier L. A. Bjork J. W. Lee A. Tranquillo R. T. (2011). Implantable arterial grafts from human fibroblasts and fibrin using a multi-graft pulsed flow-stretch bioreactor with noninvasive strength monitoring. Biomaterials 32, 714–722. 10.1016/j.biomaterials.2010.09.019 20934214 PMC3042747

[B41] Winfrey M. E. Cochran M. Hegarty M. T. (1999). A new technology in burn therapy: INTEGRA artificial skin. Dimens. Crit. Care Nurs. 18, 14–20. 10.1097/00003465-199901000-00003 10639995

[B42] Xu Y. Yao Y. Gao J. (2024). Cell-derived matrix: production, decellularization, and application of wound repair. Stem Cells Int. 2024, 7398473. 10.1155/2024/7398473 38882595 PMC11178417

